# Analysis of Pregnancy-Related Listeria monocytogenes: A Case Series and Literature Review

**DOI:** 10.7759/cureus.106355

**Published:** 2026-04-03

**Authors:** Yiying Li, Ailan Fu, Xuyang Hu, Pengju Cao, Liang Lin

**Affiliations:** 1 Gynaecology and Obstetrics, Fuzhou University Affiliated Provincial Hospital, Fuzhou, CHN; 2 Medical Centre of Maternity and Child Health, Shengli Clinical Medical College of Fujian Medical University, Fuzhou, CHN; 3 Department of Clinical Laboratory, Fuzhou University Affiliated Provincial Hospital, Fuzhou, CHN

**Keywords:** listeria monocytogenes, listeriosis, pregnancy complication, pregnancy outcome, neonatal outcome

## Abstract

Listeriosis is an infectious disease caused by Listeria monocytogenes. A retrospective analysis was conducted on four pregnant women and three newborns diagnosed and treated for listeriosis at Fuzhou University Affiliated Provincial Hospital between May 10, 2015, and June 1, 2023, using descriptive methods. Maternal Case 1 was a 33-year-old woman who underwent cesarean section at full term due to fetal distress, Case 2 was a 32-year-old woman who underwent cesarean section at preterm due to fetal distress, Case 3 was a 32-year-old woman who presented with spontaneous preterm labor, and Case 4 was a 30-year-old woman who underwent induction due to fetal malformation. Among the four pregnant women, initial symptoms included fever (n = 2), abdominal pain or vaginal bleeding (n = 3), and abnormal fetal heart monitoring (n = 2). Laboratory findings showed varying levels of procalcitonin (0.11-0.26 μg/L) and C-reactive protein (15-121 mg/L). Blood cultures of the newborns born in Cases 1 to 3 confirmed Listeria monocytogenes infection. Initial empirical treatment with cephalosporins was adjusted to penicillin, linezolid, or vancomycin based on drug susceptibility testing. Pregnancy-related listeriosis was associated with high feto-neonatal mortality. Clinicians should emphasize the importance of a healthy diet and raise awareness of this condition among pregnant women.

## Introduction

Among the genus Listeria, Listeria monocytogenes (LM) is the only species capable of causing disease in humans. Although rare, LM infections are characterized by high drug resistance and significant mortality. Pregnant women and newborns are especially vulnerable, with pregnant women facing a 10- to 20-fold increased risk of infection [1,2]. LM can be transmitted to the fetus via amniotic fluid, fetal membranes, or blood. Pregnancy-associated listeriosis may present with flu-like symptoms or remain asymptomatic, yet it can result in adverse outcomes, including fetal malformation, miscarriage, stillbirth, preterm birth, and neonatal systemic infection. The reported incidence is 13.7 cases per 100,000 pregnancies [2]. Due to limited documentation in China, obstetricians may lack sufficient awareness of this disease, leading to missed or incorrect diagnoses. This study retrospectively reviewed four cases of maternal listeriosis and three cases of neonatal listeriosis at our hospital, aiming to enhance obstetricians’ understanding of pregnancy-related listeriosis, promote early diagnosis and treatment, and improve patient outcomes.

## Case presentation

The study included four pregnant women diagnosed with pregnancy-associated listeriosis at the Fuzhou University Affiliated Provincial Hospital between May 10, 2015, and June 1, 2023. All participants presented with clinical symptoms and met at least one of the following criteria: (1) detection and isolation of LM from specimens obtained from sterile sites [3,4]; (2) onset of LM septicemia within three days postpartum. Three newborns delivered by the four aforementioned pregnant women were included. Neonatal listeriosis was defined by clinical symptoms such as fever, dyspnea, and expectoration, and met at least one of the following criteria: (1) identification and isolation of LM from sterile sites; (2) onset of LM septicemia within three days after birth [4]. This study was conducted in accordance with the Declaration of Helsinki and was approved by the Ethics Committee of the Fuzhou University Affiliated Provincial Hospital. Informed consent was obtained from each patient or their legally authorized representative if the patient was unable to provide consent.

LM identification was performed using the VITEK2 compact microbiological identification system and drug susceptibility cards from bioMérieux (Marcy l'Etoile, France). The Bactec FX200 automated blood culture system and culture bottles from Becton, Dickinson & Company (Sparks, MD, USA) were used for specimen processing. Quality control strains, including Escherichia coli and Staphylococcus aureus, were utilized. Species identification and antimicrobial susceptibility testing were conducted on isolated pure colonies using the VITEK2 system. Strains that could not be tested using the minimum inhibitory concentration (MIC) method underwent testing via the K-B method. Drug susceptibility results were interpreted according to the Clinical and Laboratory Standards Institute (CLSI) M100-S31 guidelines [5]. Clinical data collected included pregnancy complications, gestational age, mode of delivery, initial symptoms, Apgar scores, birth weight, maximum body temperature, peripheral blood white blood cell count, C-reactive protein (CRP), procalcitonin (PCT) levels, placental pathology, antibiotic regimens, duration of hospitalization, and survival outcomes.

Table 1 summarizes the clinical characteristics of four confirmed cases of pregnancy-related listeriosis among 26,689 pregnant women who delivered at the Fuzhou University Affiliated Provincial Hospital between May 10, 2015, and June 1, 2023. The incidence rate was 14.99 per 100,000. The patients were aged 30 to 33 years, and hospital stays ranged from four to 11 days. Two cases were associated with the consumption of take-out food or raw and cold fruits prior to admission. The cases occurred in April (n = 2), May (n = 1), and August (n = 1). Symptom onset ranged from 23 to 39 weeks of gestation. Three cases occurred in the third trimester (one full-term and two preterm deliveries), and one case involved induced labor in the second trimester due to "oligohydramnios and fetal malformation," resulting in a stillborn female weighing 350 g, measuring 17 cm (the case number of the mother is Case 4). All four patients experienced pregnancy complications, including gestational diabetes in three cases. Initial symptoms included fever (n = 2), abdominal pain or vaginal bleeding (n = 3), and abnormal fetal heart monitoring (n = 2).

**Table 1:** Clinical data of four pregnant women infected with Listeria monocytogenes (LM) GDM: Gestational diabetes mellitus.

Case No	Age	Gestational week	Delivery mode	Pregnancy complication	Hospitalization days	Early symptoms at onset	Amniotic fluid turbidity degree	Initial treatment	Adjusted treatment	Pregnancy outcome	Patient outcome
1	33	38+3	Conversion of natural delivery to cesarean section	GDM	6	Fever, fetal heart baseline: 170 beats/min, with variant deceleration, abdominal pain and vaginal bleeding	Degree III	Cefoperazone-Sulbactam	/	live birth	cured
2	32	34+1	Cesarean section	GDM	5	Fetal heart baseline: 170-180 beats/min, abdominal pain, vaginal bleeding	Degree III	Cefoperazone-Sulbactam	linezolid	live birth	cured
3	32	36+1	Natural delivery	Premature birth, GDM	11	Abdominal pain, vaginal bleeding	Degree III	Cefuroxime + metronidazole	Penicillin	live birth	cured
4	30	23+1	Induction of labor by Rivanol	Oligohydramnios, fetal malformation	4	Fever	Degree III	Cefuroxime	/	stillbirth	cured

Clinical course

For Case 1, symptom onset was two hours before admission with rapid progression; admission vitals were 37.6°C, heart rate (HR) 160 bpm; fetal membrane culture (+) at five days. For Case 2, symptom onset was 10 hours before admission; admission vitals were 36.6°C, HR 178 bpm; fetal membrane culture (+) at four days. For Case 3, symptom onset was two hours before admission; admission vitals were 36.5°C, HR 142 bpm; fetal membrane culture (+) at three days. For Case 4, there was intermittent fever for 20 days before admission with gradual progression; admission vitals were 36.8°C, HR 142 bpm; amniotic fluid culture (+) at five days. Culture positivity ranged from three to five days (median 4.5 days).

**Figure 1:**
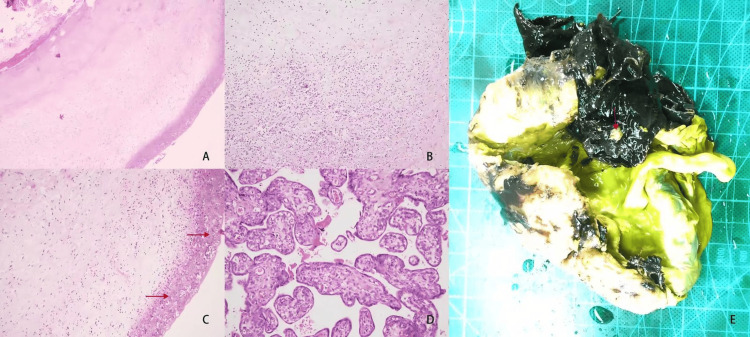
Pathological examination of placenta infected with Listeria monocytogenes (LM) (A) From Case 2, extensive infiltration of inflammatory cells in the chorioamniotic membrane (hematoxylin and eosin staining, ×40). (B) From Case 4, degeneration of placental villi and mild focal inflammatory cell infiltration (hematoxylin and eosin staining, ×100). (C) From Case 2, dense aggregation of neutrophils in the chorioamniotic membrane (arrow, hematoxylin and eosin staining, ×100). (D) From Case 4, focal infarction with fibrinoid exudation, abundant villous blood vessels (hematoxylin and eosin staining, ×200). (E) From Case 4, gross placental specimen, maternal surface scattered grayish-yellow nodules formed by abscesses within the clots (arrow).

Table 2 shows elevated levels of PCT, CRP, and WBC. One patient had a fever 20 days before admission and presented with leukopenia (1.52 × 10⁹/L), which improved after treatment with cephalosporin antibiotics. LM was isolated from the fetal membranes in three cases (culture positivity at three to five days) and from amniotic fluid in one case (positivity at five days). Blood cultures were obtained prior to antibiotic administration in all cases. Placental histopathology revealed chorioamnionitis, with abundant inflammatory cell infiltration observed in Case 4 (Figure 1). One patient had received cephalosporin antibiotics before admission. During delivery, yellow discoloration of the fetal membranes indicated possible intrauterine infection. Empirical treatment with metronidazole and cefoperazone-sulbactam was initiated. On the third postpartum day, LM was confirmed in fetal membrane cultures, and penicillin was administered. Another patient initially received cefoperazone-sulbactam, which was later switched to linezolid based on culture results. One case involved artificial membrane rupture during labor, grade III amniotic fluid, high fever, and an elevated WBC count (18.8 × 10⁹/L). Fetal heart monitoring indicated class II fetal distress. A cesarean section was performed due to suspected chorioamnionitis, and cefoperazone-sulbactam was used to prevent infection. All four pregnant women recovered and were discharged (Table 1).

**Table 2:** Laboratory findings of four pregnant women with Listeria monocytogenes (LM) infection WBC: white blood cell count; CRP: C-reactive protein; PCT: procalcitonin.

Case No	WBC (×10^9^/L）	CRP (mg/L）	PCT (μg/L）	Placental pathology	Source of LM
1	18.8	15	0.26	Not checked	Fetal membranes
2	27.8	121	Not checked	Placental chorioamnionitis	Fetal membranes
3	15	47.5	0.25	No obvious abnormality was found	Fetal membranes
4	13.4	40.6	0.11	A large number of acute and chronic inflammatory cells infiltrated and cellulose necrosis exudated in fetal membrane umbilical cord interstitium and umbilical vessel wall	Amniotic fluid

Table 3 presents the clinical data of three newborns. Symptoms developed within six days after birth. Birth weights ranged from 2540 g to 2960 g, and hospital stays ranged from three to 21 days. Major symptoms included fever (38.1-38.7 °C), dyspnea, and productive cough with yellow sputum. WBC increased in two cases and decreased in one, with a minimum value of 0.6 × 10⁹/L. CRP and PCT levels were elevated in all three cases. LM was detected in blood, cerebrospinal fluid, sputum, and cerebrospinal fluid samples. All three newborns initially received empirical cephalosporin therapy. Treatment regimens were later adjusted based on antimicrobial susceptibility testing and clinical condition. One newborn recovered and was discharged, while two died after discharge.

**Table 3:** Clinical data of three neonates infected with Listeria monocytogenes (LM) WBC: white blood cell count; CRP: C-reactive protein; PCT: procalcitonin.

	Corresponding mother	Apgar score	Maximum temperature (℃）	Clinical manifestation	WBC (×109/L)	CRP (mg/L）	PCT (μg/L)	Hospitalization days	Survival outcome	Weight (g）	Source of LM	Initial treatment	Adjusted treatment
1	Case No 1	40461	38.7	Fever, Sleepiness	0.6	15.22	9.09	4	Death	2960	Blood culture, sputum, cerebrospinal fluid	Cefotaxime	Penicillin, meropenem, vancomycin
2	Case No 2	39237	38.2	Fever, Dyspnea	20.1	＞200	53.93	21	Cure	2770	Blood culture	Cefotaxime, penicillin	Cefotaxime, linezolid
3	Case No 3	39852	38.1	Fever, cough with yellow sticky sputum	13.7	55.93	94.21	3	Death	2540	Sputum culture, gastric juice, blood culture	Cefoperazone, sulbactam	Cefoperazone, sulbactam, penicillin

## Discussion

A total of 232 articles were retrieved from PubMed using the search terms "pregnancy" and "Listeria monocytogenes" between January 2010 and August 2023. After abstract screening, the full texts of 31 articles were reviewed, and five met the inclusion criteria, reporting a total of 99 cases. After excluding 29 duplicate cases, 70 laboratory-confirmed cases, along with four cases from our hospital, were included for a total of 74 cases. Inclusion criteria allowed for pregnant individuals with non-specific flu-like symptoms and LM isolated from sterile sites. Exclusion criteria were (1) review articles without individual case reports; (2) duplicate cases from the same institution; and (3) cases with unclear diagnosis, treatment history, or incomplete data. Descriptive analysis methods were used [2,6-8].

A total of 70 laboratory-confirmed cases were analyzed, with the following observations: Listeriosis occurred in 24 cases during early or mid-pregnancy and in 46 cases during late pregnancy. Reported symptoms included lower abdominal pain (n = 30), premature rupture of membranes (n = 4), vaginal bleeding (n = 4), and abnormal fetal movement or heart rate monitoring (n = 23). LM was isolated from various sources: amniotic fluid in 12 pregnant women, blood in 33 pregnant women, placental or cervical secretions in 37 pregnant women, and blood in 22 newborns. Antibiotic regimens included cephalosporins in 36 cases. Other patients received azithromycin, piperacillin sodium, sulbactam sodium, metronidazole, amoxicillin, or clindamycin. Fetal and neonatal outcomes included 27 intrauterine deaths, 15 neonatal deaths, and 32 successful recoveries and discharges. For maternal outcomes, all mothers recovered and were discharged following treatment (Table 4).

**Table 4:** Summary of 70 cases of pregnancy-associated listeriosis in China from 2010 to 2023 The data presented in this table are compiled from multiple studies [2,6-8].

Classification		Number of cases (%)
Gestational age	＜28W	24/70（34%）
	≥28W	46/70（66%）
Clinical manifestation	Fever	53/70（76%）
	Abdominal pain	30/70（34%）
	Premature rupture of membranes	4/70（6%）
	Vaginal bleeding	4/70（6%）
	Abnormal fetal movement or fetal heart monitoring	23/70（33%）
Bacterial culture results	Amniotic fluid culture（+）	12/70（17%）
	Maternal blood culture（+）	33/70（47%）
	Placental and cervical secretion culture（+）	37/70（53%）
	Neonatal blood culture（+）	22/70（31%）
Initial treatment	Cephalosporin antibiotics	36/70（51%）
	Other antibiotics	34/70（49%）
Fetal and neonatal outcomes	Intrauterine fetal death	27/70（6%）
	Postnatal death	15/70（39%）
	Survival	32/70（46%）
Maternal outcome	Recovery	70/70（100%）
	Death	0/70（0%）

LM can cross three major physiological barriers: the intestinal epithelium, the placenta, and the blood-brain barrier. It exhibits a particular affinity for the placenta, as evidenced by placental histopathology revealing chorioamnionitis, vasculitis, and umbilical cord inflammation. In twin pregnancies, one fetus may experience intrauterine demise due to LM infection, while the co-twin may survive after postnatal treatment [9]. This discrepancy in outcomes may relate to shared placental structures in twin gestations. In the present study, LM was detected in the cerebrospinal fluid of one newborn, who developed drowsiness and other neurological symptoms, indicating central nervous system infection following transgression of the blood-brain barrier. The infant unfortunately died after discharge.

The incidence of listeriosis varies by ethnicity, socioeconomic status, and geographic location, with Asian women having a 2.3-fold higher incidence compared to European or other populations [10]. The actual incidence of listeriosis in China is likely underestimated due to insufficient surveillance and the absence of mandatory infectious disease classification, diagnostic challenges in early stages due to non-specific symptoms, and limited awareness among healthcare providers. Listeria monocytogenes is commonly found in ready-to-eat foods, including deli meats, hot dogs, soft cheeses (e.g. brie, feta, blue cheese), unpasteurized dairy products, refrigerated smoked seafood, and pre-prepared salads [11]. Pregnant women are advised to avoid these high-risk foods and to practice safe food handling, such as heating ready-to-eat foods until steaming hot and thoroughly washing raw fruits and vegetables [11]. The prevalence of LM in Chinese food has been reported at approximately 4.42% [1,2].

During pregnancy, elevated progesterone levels suppress the maternal immune response, increasing susceptibility to LM infection [12]. In this study, three of four pregnant women (75%) had gestational diabetes mellitus (GDM), a proportion notably higher than the general prevalence (8-15% in China). Pregnancy impairs cell-mediated immunity, and diabetes further compromises host defense against intracellular pathogens such as Listeria [11]. This suggests that diabetic pregnant women may represent a high-risk population warranting heightened clinical awareness and targeted food safety counseling. Further research is needed to confirm whether GDM is an independent risk factor. Fever is a common manifestation, reported in 65-81% of LM-infected pregnant women, with temperatures typically ranging from 38-39 °C [13]. In this study, both patients with recent intake of contaminated food experienced fever. A prior study involving 70 patients found that 76% had fever and 34% experienced abdominal pain, consistent with the current findings. Fever in pregnancy requires consideration of chorioamnionitis, pyelonephritis, and viral infections. In the present cases, the absence of uterine tenderness (against chorioamnionitis) and dysuria (against pyelonephritis), combined with flu-like prodrome and rapid progression to fetal distress, supported the suspicion of listeriosis, which was subsequently confirmed by culture. However, approximately 30% of patients remain asymptomatic, complicating early diagnosis [13]. Persistent LM presence in the placenta may result in recurrent infections, further challenging treatment. While LM infection can exacerbate pregnancy complications, maternal mortality is rare [14]. All mothers in this study recovered and were discharged. Emergency cesarean sections are often required due to fetal distress, occurring in approximately 82% of cases, with 55% showing meconium-stained amniotic fluid. Similarly, in this study, two patients underwent emergency cesarean sections due to fetal distress, and all three cases exhibited grade III amniotic fluid turbidity, aligning with previous reports. The lower incidence in the first and second trimesters may result from the lack of embryonic tissue sampling or maternal blood culture following miscarriage. This highlights the importance of obtaining a detailed medical history and conducting laboratory testing in cases of early pregnancy loss. All four pregnant women in this study showed elevated infection markers. However, these inflammatory indicators are non-specific, making early identification of Listeria infection difficult. In this study, pathogen cultures required three to six days, with a mean of 4.5 days, delaying initiation of targeted antimicrobial therapy. While WBC counts may rise significantly in LM-infected pregnancies, mild leukocytosis is also a physiological finding in normal pregnancy [14]. Therefore, individualized interpretation of WBC trends is necessary. Clinicians should suspect LM infection if WBC levels show a progressive increase. Notably, one patient in this study presented with fever and leukopenia upon admission, which contrasts with typical LM-related hematologic findings.

Cephalosporins are the most commonly used empirical antibiotics for infection prevention and treatment in pregnant women; however, LM exhibits resistance to this antibiotic class [15]. Experts recommend high-dose amoxicillin (over 6 g/day) for two weeks, or a combination of amoxicillin and gentamicin, as the combination of gentamicin with ampicillin or amoxicillin has been shown to improve neonatal survival. For patients allergic or resistant to penicillin, alternative treatments include trimethoprim, erythromycin, and sulfamethoxazole [15]. The standard antibiotic treatment course for LM infection is two to three weeks, but when the maternal nervous system is involved, treatment may need to be extended to four weeks [15]. The efficacy of LM vaccination and prophylactic antibiotic use remains controversial. Experts generally do not recommend preventive antibiotics in suspected LM cases, as the risk of developing invasive disease is low [16]. Accordingly, the American College of Obstetricians and Gynecologists (ACOG) advises against prophylactic antibiotics in asymptomatic pregnant women [16].

LM primarily infects newborns via transplacental transmission. A study of 166 newborns reported survival rates of 0% for those infected in early pregnancy, 29.2% in mid-pregnancy, and 95.3% in late pregnancy [17]. Neonatal listeriosis is classified as early onset disease (EOD), occurring within six days after birth, and late onset disease (LOD), occurring after six days [1,17]. LOD cases may present with severe complications such as sepsis, meningitis, growth retardation, and neurological disorders [18]. Lamont et al. concluded that premature birth, rather than LM infection itself, was the primary cause of neonatal death [19]. EOD typically manifests with respiratory distress, pneumonia, and sepsis, with a high incidence of sepsis. Approximately 40% of affected newborns may develop neurological complications within seven to 28 days postpartum [19]. In this study, all three newborns had EOD, and two died after discharge. As such, newborns should be closely monitored for neurological symptoms for two to three months after birth [14].

## Conclusions

In conclusion, clinicians should remain vigilant for LM infection in pregnant women presenting with fever, vomiting, or diarrhea, especially in cases involving twin or multiple pregnancies. For patients experiencing miscarriage in early or mid-pregnancy without typical symptoms, timely sampling of fetal membranes and cervical secretions is essential to improve diagnostic accuracy. Additionally, education for pregnant and postpartum women should be strengthened to discourage consumption of unpasteurized dairy products and ready-to-eat foods and to promote the use of clean tableware. Finally, further accumulation of clinical cases is necessary to deepen understanding of LM infection and optimize management strategies.
